# Synovial fluid lubricin increases in spontaneous canine cruciate ligament rupture

**DOI:** 10.1038/s41598-020-73270-2

**Published:** 2020-10-07

**Authors:** Yuyan Wang, David W. Gludish, Kei Hayashi, Rory J. Todhunter, Ursula Krotscheck, Philippa J. Johnson, Bethany P. Cummings, Jin Su, Heidi L. Reesink

**Affiliations:** 1 Department of Clinical Sciences, College of Veterinary Medicine, Cornell University, Ithaca, NY 14853 USA; 2 Meinig School of Biomedical Engineering, Cornell University, Ithaca, NY USA; 3 Department of Biomedical Sciences, Cornell University, Ithaca, NY USA

**Keywords:** Biomarkers, Translational research, Osteoarthritis, Animal physiology

## Abstract

Lubricin is an important boundary lubricant and chondroprotective glycoprotein in synovial fluid. Both increased and decreased synovial fluid lubricin concentrations have been reported in experimental post-traumatic osteoarthritis (PTOA) animal models and in naturally occurring joint injuries in humans and animals, with no consensus about how lubricin is altered in different species or injury types. Increased synovial fluid lubricin has been observed following intra-articular fracture in humans and horses and in human late-stage osteoarthritis; however, it is unknown how synovial lubricin is affected by knee-destabilizing injuries in large animals. Spontaneous rupture of cranial cruciate ligament (RCCL), the anterior cruciate ligament equivalent in quadrupeds, is a common injury in dogs often accompanied by OA. Here, clinical records, radiographs, and synovial fluid samples from 30 dogs that sustained RCCL and 9 clinically healthy dogs were analyzed. Synovial fluid lubricin concentrations were nearly 16-fold greater in RCCL joints as compared to control joints, while IL-2, IL-6, IL-8, and TNF-α concentrations did not differ between groups. Synovial fluid lubricin concentrations were correlated with the presence of radiographic OA and were elevated in three animals sustaining RCCL injury prior to the radiographic manifestation of OA, indicating that lubricin may be a potential biomarker for early joint injury.

## Introduction

Knee osteoarthritis (OA) is a joint disease characterized by changes in articular cartilage, subchondral bone, synovial tissues, menisci and ligaments^1^. It is a major cause of morbidity in humans and domestic veterinary patients, affecting more than 12% of U.S. population between age of 25–74 years and 20% of dogs older than one year^2,3^. Although age is a major risk factor for osteoarthritis, OA can develop in patients of all ages as a result of traumatic joint injuries, genetic pre-dispositions, or other risk factors^4^. With limited treatment options for end-stage OA, efforts have been made to understand mechanisms of OA initiation to aid early diagnosis and to help develop therapies that can delay or halt joint degeneration.

Anterior cruciate ligament (ACL) injury is one of the leading causes of post-traumatic knee OA in humans. Similarly, rupture of the cranial cruciate ligament (RCCL), the ACL equivalent in quadrupeds, is one of the major causes of OA in canine patients^3,5,6^. More than 20% of dogs over the age of one year and more than 80% of dogs over the age of eight were diagnosed with OA in one clinical study^3^, with an estimated $1.32 billion spent by U.S. pet owners on RCCL treatment in 2003^7^. RCCL is the greatest risk factor for canine stifle OA, and – as in repaired ACL human joints – surgical intervention is aimed only at joint stabilization and does not prevent OA progression following cruciate ligament rupture^8^. Medical management alone of partial or complete CCL rupture in dogs generally consists of long-term systemic nonsteroidal anti-inflammatory drug (NSAID) administration, strict exercise restriction and is often associated with lower quality of life or return to function indices when compared to surgical treatment.

Lubricin, hyaluronic acid (HA) and surface-active phospholipids are considered the major lubricating biomolecules in synovial fluid; however, how these molecules are altered in knee-destabilizing injuries is not completely understood. Lubricin, or proteoglycan 4 (PRG4), is a highly conserved glycoprotein that enables near-frictionless cartilage boundary lubrication via its extensive O-linked glycosylation^9^. Secreted by superficial zone chondrocytes and synovial fibroblasts, lubricin is found both on the articular surface and within synovial fluid^9,10^. Human patients with camptodactyly-arthropathy–coxa vara–pericarditis (CACP) syndrome who lack functional lubricin and *Prg4* knockout mice develop severe, early-onset polyarthropathy, demonstrating the significance of lubricin in chondroprotection and joint homeostasis^11–13^. In addition, lubricin-deficient synovial fluid from human patients with CAPC syndrome had inferior boundary lubricating ability, and increased joint friction was observed in mice with lubricin loss-of-function mutations^13^. The highly O-glycosylated tandem repeats give rise to lubricin’s anti-adhesive properties, which prevent aggregation of protein deposits on cartilage and inhibit synovial membrane hyperplasia^11,12^. In addition to its lubricating and anti-adhesive functions, recent studies have shown that lubricin downregulates inflammation via binding interactions with the damage associated molecular pattern receptors toll-like receptor 2 (TLR2) and toll-like receptor 4 (TLR4)^14,15^.

Despite the significance of lubricin in joint homeostasis, there is no consensus as to how synovial fluid lubricin is altered in response to joint trauma and subsequent post-traumatic osteoarthritis (PTOA). In one study investigating human patients with ACL injury, synovial fluid lubricin concentrations were acutely decreased post-injury and gradually returned to values similar to the contralateral knee over the course of a year^16^. However, elevated synovial fluid lubricin concentrations have been reported in humans with tibial plateau fracture and late-stage OA^17,18^. Similar increases in synovial fluid lubricin have also been observed in several studies in both experimentally-induced and naturally occurring PTOA models in horses^19–22^. However, studies evaluating synovial fluid lubricin in other large animal models, especially large animal models of knee joint instability, are lacking. To the authors’ knowledge, only one prior study has evaluated lubricin in a canine experimental ACL transection model, which revealed increased cartilage lubricin immunostaining depth in injured cartilage; however, synovial fluid lubricin was not measured^23^. Therefore, the objectives of this study were to measure synovial fluid lubricin concentrations in healthy canine stifle joints and in joints with spontaneous RCCL and to determine if changes in synovial fluid lubricin correlated with RCCL injury, radiographic evidence of OA, or concentrations of pro-inflammatory cytokines/chemokines in synovial fluid.

## Results

### Radiographic scoring

**Figure 1:**
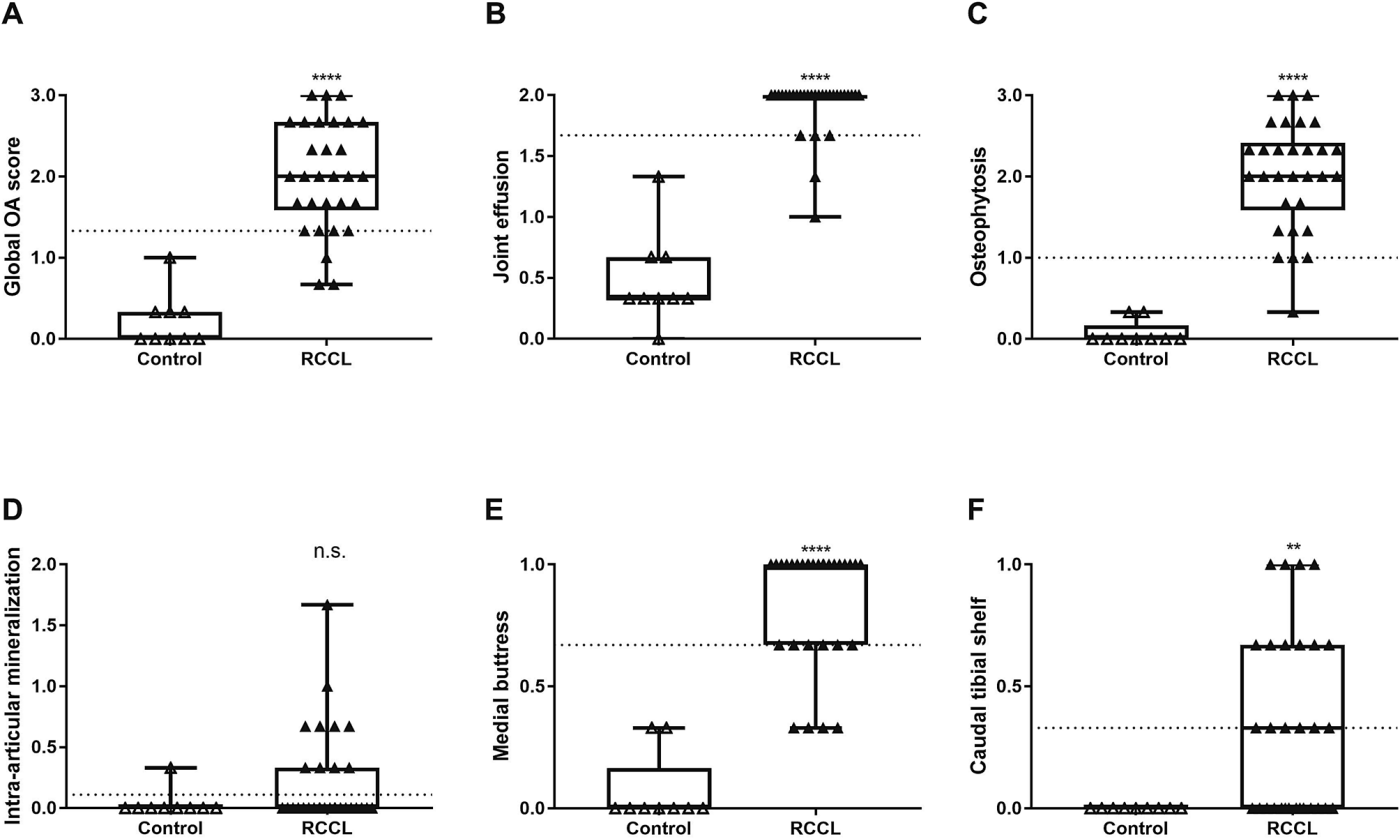
Radiographic evidence of OA in control (n = 9) and RCCL (n = 30) groups, scored by three independent observers. (**A**) Global OA score on the scale of 0–3; (**B**) joint effusion score on the scale of 0–2; (**C**) osteophytosis score on the scale of 0–3; (**D**) intra-articular mineralization score on the scale of 0–2; **E)** medial buttress score on the scale of 0–1 and (**F**) caudal tibial shelf score on the scale of 0–1. Data are displayed as box-and-whisker plots representing the first and third quartiles, median and range of concentrations for each score. Dotted line: RCCL vs. control threshold obtained by receiver operating characteristic (ROC) curve. ** *p* < 0.01, *** *p* < 0.001, **** *p* < 0.0001 for Wilcoxon test, α = 0.05.

Dogs with RCCL had significantly higher radiographic OA scores as compared to controls, with the exception of intra-articular mineralization (Fig. 1, Supp. Figure 1). Objective, blinded assessment of stifle radiographs revealed that all but three of the RCCL subjects were assigned a radiographic global OA score above the threshold obtained by receiver operating characteristic (ROC) curve analysis (Figs. 1A, 3A). Further examination revealed that these three subjects with low global OA scores had short durations of injury at the time of synovial fluid collection (t = 1, 5 and 30 days), suggesting that radiographic evidence of OA had yet to develop following RCCL injury. Linear weighted kappa tests showed fair to good inter-rater agreements for the six radiographic parameters. Between one board-certified veterinary radiologist (PJ) and one board-certified veterinary surgeon (HR), there was good agreement for global OA, joint effusion, osteophytosis and caudal tibial shelf scores, and moderate agreement for intra-articular mineralization and medial buttress scores (Supp. Table 1).

**Table 1:** Canine synovial fluid inflammatory cytokine/chemokine concentrations in control (n = 8) and RCCL (n = 28) groups.

Cytokine/chemokine	Control (pg/mL)	RCCL (pg/mL)
IL-2	2.0 ± 0.2	1.9 ± 0.3 (n.s.)
IL-6	429.4 ± 237.3	453.1 ± 179.4 (n.s.)
IL-8	493.0 ± 108.0	396.9 ± 93.1 (n.s.)
TNF-α	1.0 ± 0.5	0.3 ± 0.1 (n.s.)

Data reported as mean ± SEM, n.s.: not significant, Wilcoxon test, α = 0.05.

### Synovial fluid lubricin concentrations

**Figure 2:**
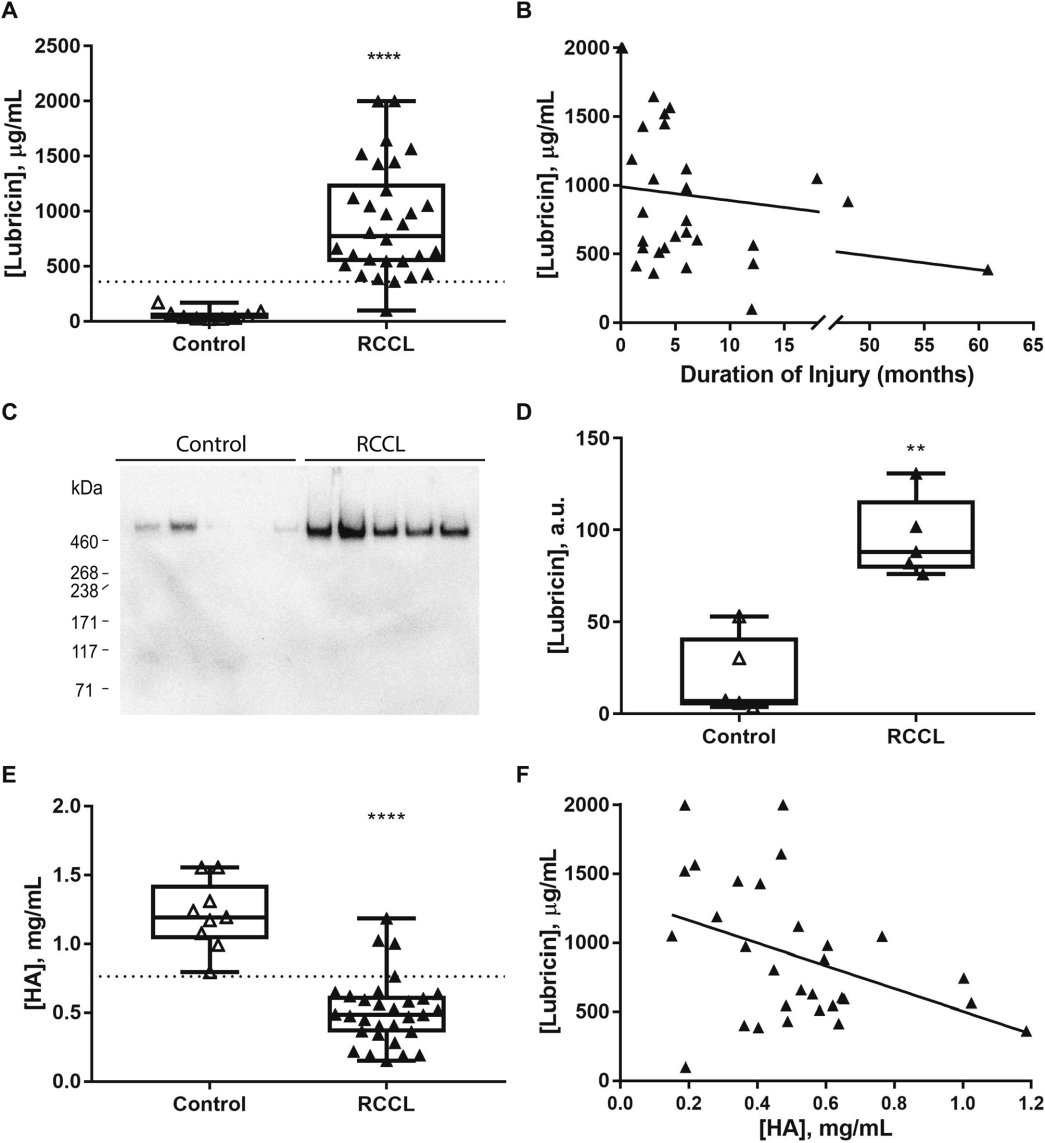
Canine synovial fluid lubricin and HA quantification. (**A**) Canine synovial fluid lubricin concentrations in control (n = 9) and RCCL (n = 30) joints. Dotted line: RCCL vs. control threshold (361.72 µg/mL) obtained by ROC curve. (**B**) Synovial fluid lubricin concentration plotted as a function of injury duration in dogs with RCCL (n = 30), solid line: R^2^ = 0.07, Spearman’s correlation test ρ = -0.35, n.s. **C)** Anti-lubricin (mAb 9G3, MABT401) western blots of synovial fluid from 5 randomly selected control and RCCL dogs. (**D**) Quantification of western blot in part C, reported as absorbance units (a.u.). (**E**) Synovial fluid HA concentration, dotted line: RCCL vs. control threshold (0.76 mg/mL) obtained by ROC curve. (**F**) Synovial fluid lubricin vs. HA concentrations for dogs with RCCL, R^2^ = 0.17, Spearman’s correlation test ρ = -0.41, *p* = 0.03. ** *p* < 0.01, **** *p* < 0.0001 for Wilcoxon test, α = 0.05.

**Figure 3:**
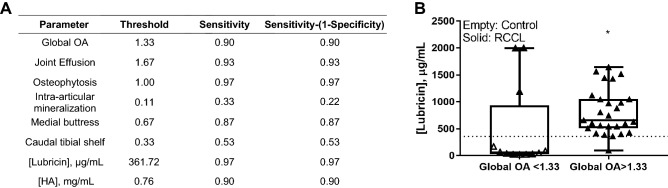
(**A**) Parameter thresholds obtained from the ROC curve analysis. (**B**) Synovial fluid lubricin concentration plotted as a function of radiographic global OA score greater than or less than 1.33. Dotted line: RCCL vs. control threshold of synovial fluid lubricin (361.72 µg/mL) obtained by ROC curve. * *p* < 0.05 for Wilcoxon test, α = 0.05.

The intra-assay coefficient of variation for the lubricin ELISA was 11.6%, and 1 sample was assigned a value of 2000 µg/mL, or the upper limit of detection of the assay due to oversaturation. The mean synovial fluid lubricin concentration in the RCCL group was 905.2 ± 91.8 µg/mL (mean ± SEM), which was approximately 16 times greater than the control group (58.7 ± 16.1 µg/mL, mean ± SEM) (Fig. 2A, p < 0.0001). Synovial fluid lubricin levels were elevated for up to 60 months post-injury (Fig. 2B), and duration of injury did not correlate with lubricin concentration. Western blot analysis showed that the molecular weight of measured lubricin is approximately 460 kDa, consistent with highly glycosylated lubricin. The mean intensity for the five selected RCCL samples was 95.6 ± 9.8 absorbance units (a.u., mean ± SEM) as compared to 19.9 ± 9.6 a.u. (mean ± SEM) for controls (Fig. 2C-D, Supp. Fig. 3). The elevated synovial fluid lubricin concentration was also correlated with the presence of radiographic OA (Fig. 3B).

### Synovial fluid HA concentrations

The intra-assay coefficient of variation for the HA ELISA was 3.17%. The mean synovial fluid HA concentration in the control group was 1.21 ± 0.08 mg/mL, and the HA concentration in the RCCL group was 0.52 ± 0.05 mg/mL (mean ± SEM) (Fig. 2E, p < 0.0001). Synovial fluid lubricin levels were inversely correlated with synovial fluid HA levels in dogs with RCCL (Fig. 2F, p = 0.03).

### Canine synovial fibroblast adhesion assay

**Figure 4:**
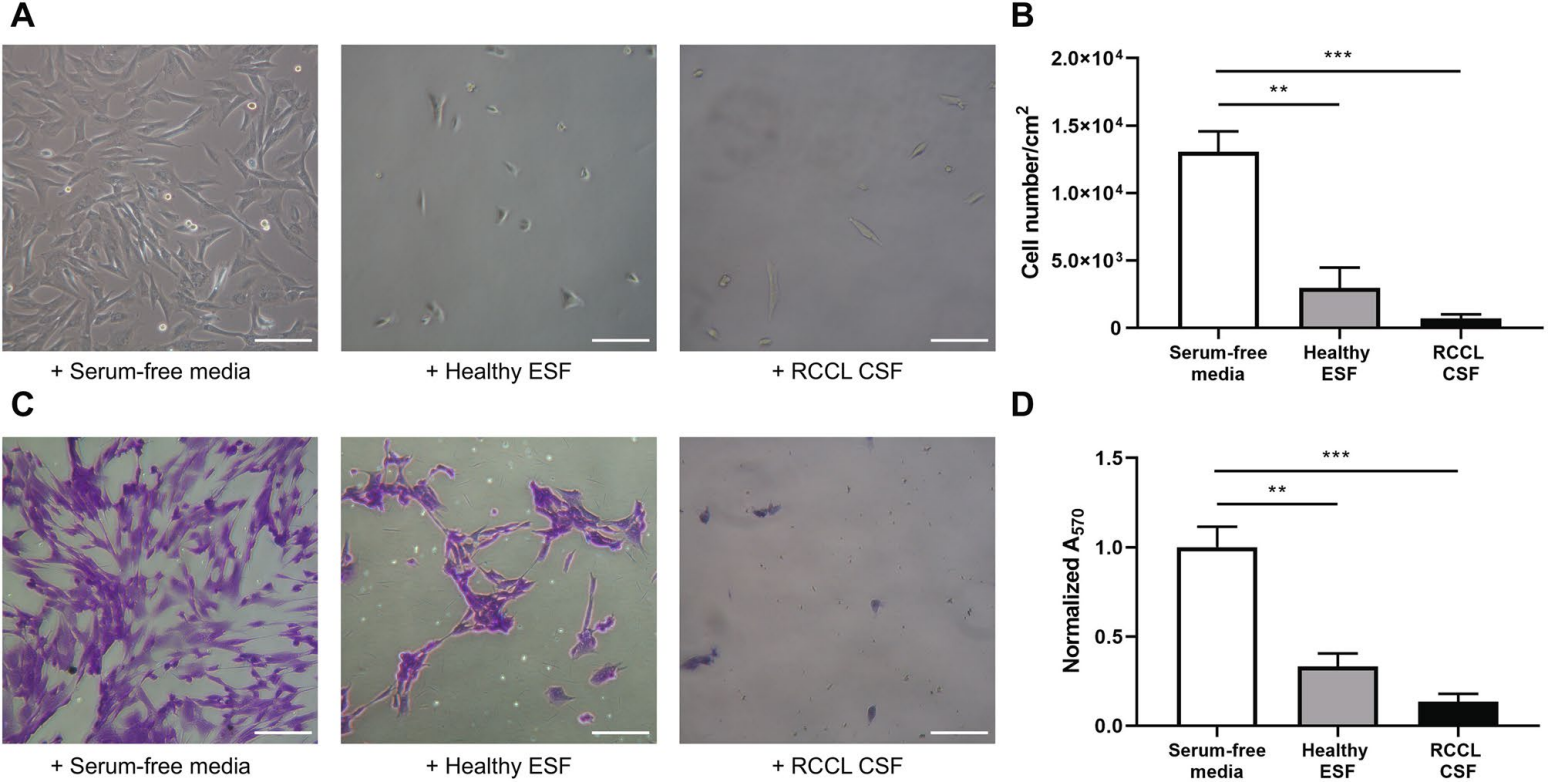
Anti-adhesive effect of RCCL canine synovial fluid (n = 4) and healthy equine synovial fluid (n = 2) pre-treatment on two primary canine synovial fibroblast cell lines. (**A**) Representative images and (**B**) average cell number after 24 h culture in serum-containing media. (**C**) Representative images of crystal violet-stained cells after 72 h, and (**D**) absorbance at 570 nm normalized to the serum-free media-treated negative control. Scale bar: 100 µm. **p < 0.01, ***p < 0.001 for *post-hoc* Dunnett's test, α = 0.05.

At 24 h, synovial fibroblast adhesion was reduced in the RCCL canine synovial fluid-treated wells (370 ± 156 cells/cm^2^) and healthy equine synovial fluid-treated wells (3.0 × 10^3^ ± 1.5 × 10^3^ cells/cm^2^) as compared to the serum-free media wells (1.3 × 10^4^ ± 1.5 × 10^3^ cells/cm^2^) (Fig. 4 A,B; data reported as mean ± SEM). At 72 h, synovial fibroblast adhesion assessed by crystal violet staining was significantly reduced in both RCCL canine synovial fluid-treated wells (13.5 ± 4.4%) and healthy equine synovial fluid-treated wells (33.1 ± 7.4%) as compared to serum-free media (Fig. 4 C,D; data reported as A_570_ normalized to the serum-free media negative control, mean ± SEM). The anti-adhesive effect of RCCL canine synovial fluid on primary canine synovial fibroblasts is comparable to that of healthy equine synovial fluid.

### Synovial fluid cytokine concentrations

Due to limited sample volume, 28 of 30 RCCL synovial fluid samples and 8 of 9 control synovial fluid samples were analyzed for cytokine/chemokine concentrations. The intra-assay coefficients of variation for IL-2, IL-6, IL-8 and TNF-α were 9.2%, 6.7%, 9.6%, and 20.5%, respectively. No significant differences were observed in cytokine or chemokine levels between control and RCCL groups (Table 1). All four cytokines/chemokines were correlated with one another (*p* < 0.05), except for IL-2 and IL-6 (ρ = 0.31 with *p* = 0.11). Lubricin concentrations did not correlate with IL-2, IL-6, IL-8, or TNF-α (Supp. Table 2). None of the four cytokines/chemokines measured were correlated with injury duration (Supp. Table 2, Supp. Figure 2).

## Discussion

RCCL injury is one of the most common orthopedic injuries in dogs and often results in stifle OA despite surgical interventions^6,24^. While the exact pathology of canine RCCL remains unknown, it is recognized as either the result of traumatic injury to the CCL^24^ or the result of degenerative CCL disease that eventually leads to ligament rupture secondary to a non-contact injury, similar to that found in female athletes^6^. Prior studies evaluating naturally occurring RCCL in client-owned dogs have shown increased pro-inflammatory cytokines, matrix metalloproteases, and lymphocytes within the joint^25–27^. Increased depth of cartilage lubricin staining was also reported in dogs with experimentally induced RCCL^23^; however, synovial fluid lubricin was not measured in that study and has not been previously measured in dogs with spontaneous RCCL.

Here, synovial fluid lubricin concentrations were approximately 16-fold greater in RCCL joints as compared to healthy stifle joints. Both RCCL injury and synovial fluid lubricin concentrations correlated with the presence of radiographic OA. No correlations were observed between synovial fluid lubricin concentrations and duration of injury. Synovial fluid HA concentrations were approximately 2.5-fold less in RCCL joints as compared to healthy stifle joints, and lubricin and HA concentrations were inversely correlated. Synovial fluid IL-2, IL-6, IL-8 and TNF-α concentrations did not differ between RCCL and control groups, perhaps due to the extended durations of injury prior to presentation. The changes in synovial fluid composition in RCCL joints did not affect the anti-adhesive properties of the RCCL synovial fluids which are largely attributed to lubricin^12^.

Elevations in synovial fluid lubricin were observed as early as one day post-injury and, in some cases, persisted for more than one-year post-injury. Three dogs from the RCCL group had minimal evidence of radiographic OA but high synovial fluid lubricin concentrations. Further examination revealed that these three animals presented within 30 days of RCCL injury, suggesting that elevations in synovial fluid lubricin preceded the development of radiographic OA. Prior studies have shown that mechanical changes in articular cartilage can precede biochemical changes^23,28^, and increased dynamic shear stress and compression of superficial zone cartilage has been shown to result in a rapid increase in lubricin production^29,30^. Joint laxity is considered to be an initiating event for precipitating OA, and ACL/CCL injuries often lead to knee destabilization^31,32^. In human OA, synovial fluid lubricin was positively correlated with anteroposterior laxity, full flexion angle, and range of knee motion^33^. Therefore, it is possible that the altered mechanical loading after ACL/CCL rupture is a stimulus for increased lubricin production as observed in canine RCCL. We hypothesize that lubricin/PRG4 may serve as one of the first responders to joint mechanical instability and increased cartilage shear stress induced by injuries such as RCCL; however, this mechanism remains to be investigated. Nonetheless, the elevated synovial fluid lubricin concentrations observed at 1-, 5-, and 30-days following RCCL injury suggest that lubricin may have potential as a biomarker for early joint injury or instability. In fact, lubricin has been shown to be a crucial component of a biomarker panel predicting radiographic progression of knee OA in human patients, with an increase in plasma lubricin concentration correlating with joint space narrowing^34^.

Decreased synovial fluid lubricin concentrations have been reported in one study evaluating human ACL injury^16^, however, in tibial plateau fracture and end-stage OA, synovial fluid lubricin concentrations are elevated^17,18,34^. There is a lack of consensus about how lubricin is altered across injury types in both human in animal studies. In experimentally-induced knee destabilization injuries in small laboratory animal models, such as rodents, guinea pigs and rabbits, decreased synovial fluid lubricin and cartilage lubricin staining are commonly reported^35–38^. As compared to rodent and rabbit models, synovial fluid acquisition is more reliable in large animal models such as the dog, sheep, and horse, where synovial fluid can be directly aspirated without the requirement for joint lavage. Interestingly, elevated synovial fluid lubricin has been observed in both experimental and naturally occurring PTOA in horses^19–22,39^, similar to that observed in spontaneous canine RCCL in the current study.

To date, every study that has evaluated lubricin supplementation in an experimental animal model of PTOA has demonstrated a benefit^40^; however, the majority of these studies are limited to rodents, with one study evaluating lubricin in a Yucatan mini-pig model^41^. Whether intra-articular lubricin supplementation will be beneficial for treating patients with increased endogenous synovial fluid lubricin concentrations in response to injury remains to be investigated. Lubricin’s beneficial effects have been primarily attributed to its anti-adhesive and boundary lubricating functions on the articular cartilage surface, yet new evidence is accumulating to suggest that lubricin also has biological functionality^9,14,15,42^. Lubricin has recently been shown to inhibit NF-κΒ-mediated inflammation by binding to CD44, TLR2, and TLR4^14,15,43^, and murine synovial *Prg4* + progenitor cells have been demonstrated to migrate to fill articular cartilage injury sites^44^. In our study, we observed reduced canine synovial fibroblast cell adhesion by canine RCCL synovial fluid, similar to anti-adhesive effects of healthy equine synovial fluid. The highly *O-*glycosylated tandem repeats give rise to lubricin’s anti-adhesive and boundary lubricating properties, which prevent aggregation of protein deposits on cartilage and inhibit synovial membrane hyperplasia^11,12,45^. Lubricin’s glycosylation varies in disease, with altered sialylation of Core 1 *O-*glycans and reduced levels of Core 2 *O-*glycans in equine joints with OA lesions and osteochondral fragmentation as compared to normal joints^46^ and increased sialylation of Core 1 *O-*glycans in human rheumatoid arthritis as compared to OA^47^. Even if lubricin is not degraded or fragmented in joint disease, changes in glycosylation may be affecting its physical and biological functionality, which might be restored by supplementation with lubricin with a healthy glycoprofile.

Synovial fluid HA concentrations were approximately 2.5-fold less in RCCL joints, while lubricin concentrations were nearly 16-fold greater compared to controls. Lubricin and HA concentrations were inversely correlated. These findings suggest that distinct mechanisms may be responsible for regulating HA and PRG4/lubricin levels in OA; however, the factors leading to increased synovial fluid lubricin in joint disease are still unknown. Lubricin and HA function synergistically in joint and cartilage lubrication^48–51^. Lubricin has been shown to crosslink HA in synovial fluid to form an elastic gel-like complex that enables the synovial fluid network to slowly dissipate strain energy, achieving a chondroprotective effect distinctive from the boundary lubrication^50^. In vitro rheological studies demonstrated that supplementing lubricin to PBS solutions with lower HA concentrations resulted in increased viscosity and shear-thinning strength^51^. Lubricin also improves wear protection of chemically-grafted HA on mica surfaces^49^. It has been hypothesized that lubricin may be elevated to compensate for the loss of HA by restoring cartilage lubrication after joint injury^39^.

In contrast to lubricin and HA, there were no differences in pro-inflammatory cytokine and chemokine concentrations in synovial fluid observed between the RCCL and control groups. The lack of differences observed in cytokines/chemokines between groups may be influenced by the administration of systemic anti-inflammatory medications prior to joint sampling and by the variation in duration of injury between patients. There is significant variability among previously reported quantities or activities of inflammatory mediators in canine RCCL joints, results which may vary with the method of bioassay used to quantify these cytokines. For example, while TNF-α is known to be increased in human joints with ACL rupture even in the chronic phase, one prior canine arthritis study found no such increases using a TNF-α bioassay, while a second study demonstrated large increases in TNF-α activity^52,53^. These data highlight the impact that sampling and assay methodology likely have on quantification of inflammatory mediators in the post-injury joint, even in prospective studies.

Limitations of this study include the retrospective study design which limited synovial fluid sample collection to a single timepoint and the variation in duration of injury prior to presentation; however, the variation in duration of injury allowed us to demonstrate that synovial fluid lubricin remains elevated for an extended period of time post-injury. In addition, the control population was limited to intact male Beagles due to the inability to harvest synovial fluid from healthy, client-owned dogs. However, considering that CCL disease is often bilateral in canine patients, the synovial fluid samples obtained from control animals without radiographic evidence of OA had advantages as compared to using synovial fluid from the contralateral limb.

Spontaneous RCCL is a common orthopedic condition in dogs that, similar to humans, frequently leads to PTOA. A near 16-fold increase in synovial fluid lubricin concentration was observed in the RCCL group as compared to controls, whereas none of the cytokines/chemokines measured correlated with RCCL injury. We observed a similar pattern of elevated synovial fluid lubricin in spontaneous canine RCCL as to what has been observed in equine osteochondral fragmentation, human intra-articular fracture, and human late-state OA.

## Methods

### Ethics statement

All sample collection protocols were approved by the Cornell University Institutional Animal Care and Use Committee (Protocol #2005–0151 and Protocol #2015–0017), and all methods were carried out in accordance with approved guidelines.

### Sample selection

Knee (stifle joint) synovial fluid samples from 30 adult dogs sustaining rupture of the cranial cruciate ligament and presenting to the Cornell University Hospital for Animals from 2014 to 2018 were obtained with informed consent at the time of surgery and stored in liquid nitrogen (Supp. Table 3). Medical histories, physical examination findings, and surgical data were obtained from patient medical records, including breed, sex, age, and duration of injury prior to synovial fluid collection. Knee joint synovial fluid samples were also obtained from a cohort of 9 clinically healthy control dogs without radiographic evidence of OA. Although bilateral samples were obtained from the healthy animals, only one, randomly assigned joint was analyzed per dog to fulfill assumptions of independence for statistical modeling.

### Radiographic scoring

Pre-operative or baseline mediolateral and craniocaudal stifle radiographs were randomized, blinded and scored by a panel of three independent observers composed of one board-certified radiologist (PJ), one veterinary surgeon (HR), and one veterinary student researcher (DG). A stifle OA scoring system adopted from Innes et al. was used, which evaluated four parameters including joint effusion (0–2), osteophytosis (0–3), intra-articular mineralization (0–2), and a global OA score (0–3) with 0 being no evidence and 2 or 3 being most severe^54^. Presence of medial buttress and caudal tibial shelf were also evaluated on a binary scale, with 0 being absence and 1 being presence of radiographic signs.

### Lubricin, inflammatory cytokine/chemokine, and HA quantification

For the lubricin ELISA, all canine synovial fluid supernatants were diluted 1:2,000 in DPBS, aliquoted and stored at -70 °C for up to 2 weeks. A custom sandwich enzyme-linked immunosorbent assay (ELISA) was used to measure synovial fluid lubricin, similar to previously described, but with a purified bovine synovial fluid lubricin standard^19^. Transparent 96-well plates (Corning, Corning, NY) were coated with 10 μg/mL of peanut agglutinin (Cat #: L0881, MilliporeSigma, Burlington, MA) in 50 mM sodium bicarbonate buffer at pH 9.5 and incubated at 4 °C overnight. The plates were blocked with 3% bovine serum albumin (VWR, Radnor, PA) in DPBS for 1 h at room temperature. Bovine synovial fluid (Cat #: 8,600,853, Lampire Biological Laboratories, Pipersville, PA) was centrifuged at 4,000 × g for 1 h at 4 °C to remove red blood cells, followed by incubation with Streptomyces hyaluronidase and protease inhibitor cocktail in 50 mM NaAc overnight at 4 °C. The synovial fluid was purified using DEAE Sepharose (Cat #: 17,070,901, GE Healthcare Bio-Sciences, Pittsburgh, PA) anion exchange fast liquid protein chromatography (FPLC) similar to previously described methods, and the 490–570 mM NaCl elution fraction was used as the standard for the lubricin ELISA^19^. The standard ladder of 12 concentrations of purified bovine lubricin ranged from 0.002 µg/mL to 1 µg/mL, in increments of two-fold serial dilutions. Diluted canine synovial fluid samples and FPLC-purified, bovine synovial lubricin were added to the plates in duplicate and incubated for 1 h at room temperature with gentle shaking. A 1-h incubation with anti-lubricin monoclonal primary antibody (Cat #: MABT401, MilliporeSigma, Burlington, MA) diluted 1:5,000 in DPBS was followed by a 1-h incubation with secondary antibody (Cat #: AP126P, MilliporeSigma, Burlington, MA) diluted 1:2,000 in DPBS^28^. Both incubations were done at room temperature with gentle shaking. The plates were developed at room temperature with 1-Step Ultra TMB (Cat#: 34,028, Thermo Fisher Scientific, Waltham, MA) for 20 min or until a royal blue color appeared, at which point the reaction was stopped with 2 N H_2_SO_4_. Absorbance was measured at 450 nm with 540 nm background subtraction on a microplate reader (Spark 10 M, Tecan, Männedorf, Switzerland), and concentrations were calculated using SparkControl Magellan 2.2 (https://lifesciences.tecan.com/software-magellan, Tecan, Männedorf, Switzerland) with a four parameter Marquardt fit. Inflammatory cytokines and chemokines, including IL-2, IL-6, IL-8, and TNF-α were measured in undiluted canine synovial fluid samples using a canine-specific electrochemiluminescent multiplex immunoassay (Cat #: K15035C-1, Meso Scale Diagnostics, Rockville, MD) following the manufacturer instructions^55–57^. Synovial fluid HA concentrations were measured using a commercially available sandwich ELISA (R&D Systems, Minneapolis, MN). Synovial fluid samples were diluted 1:80,000 in PBS with 5% Tween20, and HA concentrations were measured in duplicate according to manufacturer instructions. Absorbance was measured at 450 nm with 540 nm background subtraction.

### Canine synovial fibroblast adhesion assay

Primary canine synovial fibroblasts were isolated from the right hips of two dogs undergoing total hip replacement surgeries (3 yo. castrated male German Shepherd; 5 yo. spayed female Labrador Retriever). Briefly, canine synovial membrane tissue was placed in a petri dish in Dulbecco’s Modified Eagle’s Medium with 1 g/L glucose, 4 mM L-glutamine, 110 mg/L sodium pyruvate (DMEM/Low Glucose) (Thermo Fisher Scientific, Waltham, MA), supplemented with 25 mM HEPES buffer, 10% fetal bovine serum, 100 IU/mL penicillin and 100 µg/mL streptomycin. The tissue was then cut into pieces no larger than 0.5 cm. Dissected tissue was digested with 10 mL/g growth media containing 1.5 g/L collagenase Type II (Worthington Biochemical, Lakewood, NJ) and 0.15 g/L DNase I (Roche, Branchburg, NJ) at 37 °C for 2 h with agitation. The digested tissue was then filtered through cheesecloth and 41 μm nylon mesh (MilliporeSigma, Burlington, MA) and centrifuged at 400 × g for 10 min at room temperature. Cell pellets were re-suspended in growth media and plated at 5 × 10^4^ cells/cm^2^. Primary canine synovial fibroblasts were culture-expanded and stored as passage 0 (P0) in liquid N_2_ cryotanks prior to the experiment. P0 cells were plated and grown in growth media for an additional 2 passages prior to plating as P3 cells for adhesion assays.

Synovial fluid from the middle carpal joints of two healthy horses were used as healthy control synovial fluid due to the limited volumes of healthy canine synovial fluid available. Four canine synovial fluid samples with volumes greater than 0.3 mL were randomly selected from the RCCL group. All synovial fluid samples were diluted to 12.5% in serum-free DMEM/low glucose media. In each 24-well tissue culture plate (Greiner Bio-One, Monroe, NC), the bottom of duplicate wells were coated with 0.5 mL of serum-free medium (n = 4), 12.5% healthy equine synovial fluid (n = 2) or 12.5% RCCL canine synovial fluid (n = 4) in serum-free medium for 24 h at 37 °C. The wells were washed 3 times with DPBS prior to seeding with 45,000 cells per well (1.9 cm^2^) in 0.5 mL serum-containing medium and cultured at 37 °C and 5% CO_2_. Two primary canine primary synovial fibroblast cell lines were cultured in two separated plates, and data were reported as mean values for the combined two cell lines. After 24 h, the cells were imaged with an Olympus CK2 inverted microscope (150 × magnification) and Nikon DS-Fil camera. In addition, five images per well (center and four corners) were captured with a microplate reader (Spark 10 M, Tecan, Männedorf, Switzerland) for cell counting in Image J. Mean cell number was calculated using 5 fields/well. After an additional 48 h incubation at 37 °C, the cells were stained with 0.4 mL of 0.5% crystal violet solution in 20% methanol for 20 min at room temperature. Phase contrast images of stained cells were captured at 150 × magnification, and the absorbance at 570 nm was measured with a microplate reader. All absorbance measurements were normalized to the average A_570_ of the serum-free media negative control group.

### Western blots

Five control and five RCCL canine synovial fluid samples were randomly selected and diluted 1:1,000 in DPBS. 10 μL of each sample was loaded onto and separated on NuPAGE 3–8% Tris–Acetate gels (Invitrogen, Carlsbad, CA), followed by transfer to a PVDF membrane (MilliporeSigma, Burlington, MA). The membrane was blocked with 5% skim milk powder in PBS with 0.1% Tween20 for 1 h at room temperature. The anti-lubricin primary antibody (Cat #: MABT401, MilliporeSigma, Burlington, MA) was diluted 1:1,000 in half-strength blocking buffer, and the membrane was incubated overnight at 4 °C. The secondary antibody (Cat #: AP126P, MilliporeSigma, Burlington, MA) was diluted 1:10,000 in PBS with 0.1% Tween20 and incubated for 1 h at room temperature. The immunoblot was developed in enhanced chemiluminescence substrate (Bio-Rad Laboratories, Hercules, CA) and imaged on a ChemiDoc (Bio-Rad Laboratories, Hercules, CA) imaging system. The signal intensities were analyzed using ImageJ. 

### Statistical analysis

Investigators were blinded to radiographic or sample status for all outcome measures. Normality of each sample distribution was assessed prior to statistical analysis with the Shapiro–Wilk test (α = 0.05). For radiographic data, linear weighted kappa tests were used to assess the inter-rater agreement, with kappa values < 0.20 being poor, 0.21–0.40 being fair, 0.41–0.60 being moderate, 0.61–0.80 being good, and 0.81–1.0 being very good. Control subjects with global OA scores > 1 were excluded from analysis as these were not considered healthy controls due to the radiographic presence of OA.

Thresholds to discriminate between control and RCCL groups for radiographic parameters and synovial fluid lubricin and HA concentrations were obtained from the value that maximized sensitiviy-(1-specificity), or the Youden Index, on the receiver operating characteristic curve. An ANOVA test (α = 0.05), followed by *post-hoc* Dunnett’s test (α = 0.05) was used for cell count and absorbance value in the cell adhesion assay with the serum-free media treatment considered the control. Wilcoxon rank-sums tests (α = 0.05) were used to analyze differences between all other data sets, including radiographic OA grades and synovial fluid lubricin, HA, and cytokine concentrations. Spearman’s rank-order correlations were performed to determine associations between injury duration, lubricin concentration, and inflammatory cytokine/chemokine concentrations. A *p* value < 0.05 was considered significant. The linear weighted kappa test was performed in MedCalc 18.10 (MedCalc Software, Ostend, Belgium), and the remaining statistical analyses were performed in JMP Pro 13 (SAS Institute, Cary, NC).

## Supplementary information

Supplementary information

Supplementary information

## Data Availability

The datasets generated during and/or analysed during the current study are available from the corresponding author on reasonable request.
