# Supplementary material for: Synovial fluid lubricin increases in spontaneous canine cruciate ligament rupture
Source: Sci Rep. 2020 Oct 7;10:16725. doi: 10.1038/s41598-020-73270-2 (PMC7542452; doi:10.1038/s41598-020-73270-2)
Supplement: Supplementary file 1 — Supplementary information [file 41598_2020_73270_MOESM1_ESM.pdf]

Synovial Fluid Lubricin Increases in Spontaneous Canine Cruciate Ligament Rupture

Yuyan Wang[1, 2]†, David W. Gludish[1]†, Kei Hayashi[1], Rory J. Todhunter[1], Ursula Krotscheck[1], Philippa J. Johnson[1], Bethany P. Cummings[3], Jin Su[1], Heidi L. Reesink[1*]

[1]Department of Clinical Sciences, College of Veterinary Medicine, Cornell University, Ithaca, NY

[2]Meinig School of Biomedical Engineering, Cornell University, Ithaca, NY

[3]Department of Biomedical Sciences, Cornell University, Ithaca, NY

†Both authors contributed equally to this manuscript

***Please Address Correspondence to:**

Heidi Reesink VMD PhD

Cornell University College of Veterinary Medicine

VMC C3-101

Ithaca, NY 14853

Phone: 607-253-3100

Email: hlr42@cornell.edu

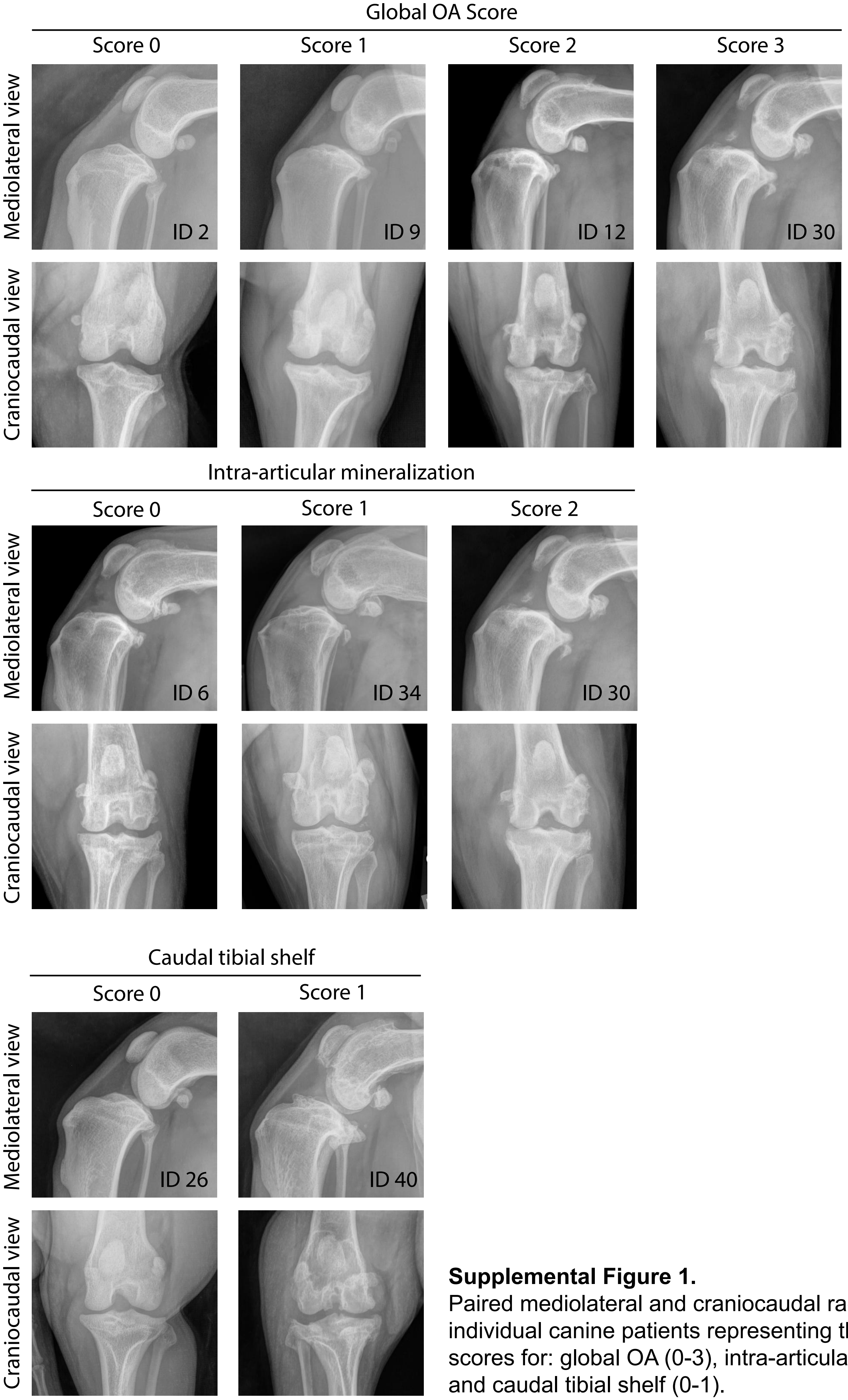

Supplemental Figure 1.
Paired mediolateral and craniocaudal radiographs from individual canine patients representing the range of possible scores for: global OA (0-3), intra-articular mineralization (0-3) and caudal tibial shelf (0-1).

	PJ vs. HR	HR vs. DG	PJ vs. DG
**Global OA score**	0.659	0.674	0.312
**Joint effusion**	0.736	0.567	0.502
**Osteophytosis**	0.712	0.712	0.700
**Intra-articular mineralization**	0.493	0.396	0.393
**Medial buttress**	0.433	0.570	0.474
**Caudal tibial shelf**	0.692	0.665	0.479

Supplemental Table 1. Linear weighted Kappa interrater agreement for canine radiographic osteoarthritis scoring. Strength of agreement was interpreted with kappa values: 0-0.2 being poor, 0.21-0.40 being fair, 0.41-0.60 being moderate, 0.61-0.80 being good, and 0.81-1.00 being very good for interrater agreement between scores for three co-authors, including the second (DG), sixth (PJ) and final co-author (HR).

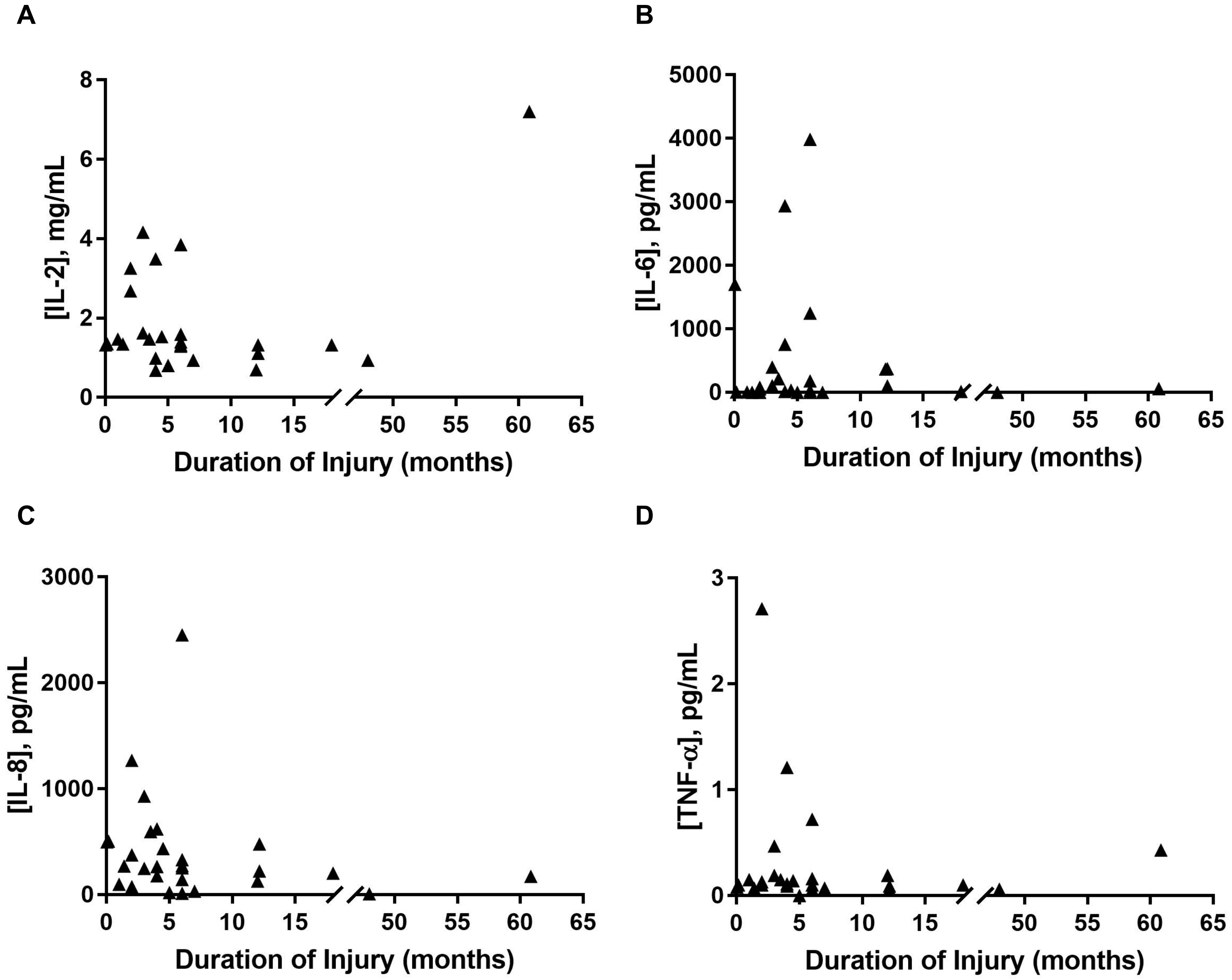

Supplemental Figure 2. Synovial fluid A) IL-2, B) IL-6, C) IL-8, and D) TNF-α concentrations plotted as a function of injury duration in dogs with RCCL (n = 28).

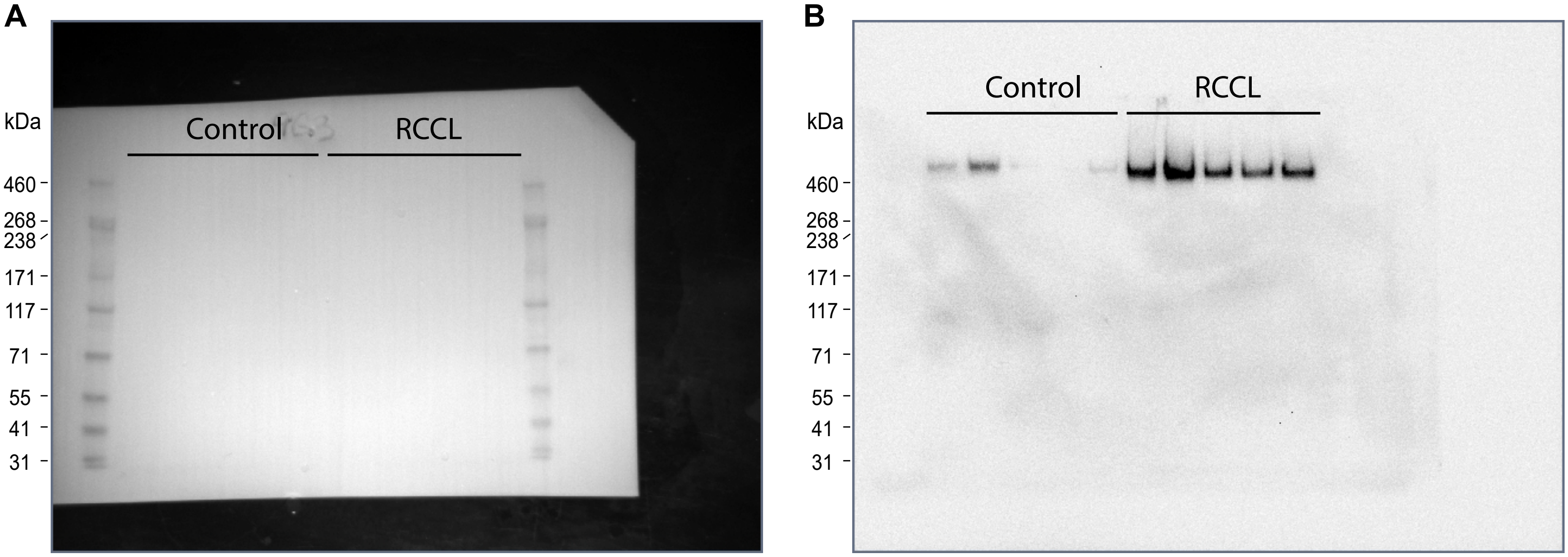

Supplemental Figure 3. Anti-lubricin western blots of synovial fluid from 5 randomly selected control and RCCL dogs. A) Bright field image of standard ladders (HiMark™ Pre-stained Protein Standard, cat. #LC5699, Invitrogen), 5 μL/lane, B) chemiluminescence, exposure time: 15 s; sample loaded:10 μL/lane.

Cytokine/Chemokine	Lubricin	Duration of Injury
IL-2	-0.23 (n.s.)	-0.31 (n.s.)
IL-6	-0.24 (n.s.)	-0.02 (n.s.)
IL-8	-0.11 (n.s.)	-0.39 (n.s.)
TNF-α	-0.25 (n.s.)	-0.17 (n.s.)

Supplemental Table 2. Correlation of inflammatory cytokine/chemokine concentrations, lubricin concentrations and injury duration in dogs with RCCL (n = 28). Data reported as Spearman's ρ, n.s.: not significant, Spearman's test, α = 0.05.

	Control	RCCL
**Number**	N = 9	N = 30
**Age**	3.3 - 7.6 yrs, mean = 5.5 yrs	2.6 – 11.0 yrs, mean = 6.2 yrs
**Sex**	Intact male	13 neutered male<br>14 spayed female<br>2 intact male<br>1 intact female
**Breed**	Beagle	1 Bernese Mountain Dog<br>1 Boxer<br>1 Bulldog, English<br>1 Cane Corso<br>2 English Springer Spaniel<br>1 German Shepherd<br>3 Golden Retriever<br>9 Labrador Retriever<br>1 Mastiff<br>6 Mixed Dog<br>1 Pembroke Welsh Corgi<br>2 Rottweiler<br>2 Staffordshire Bull Terrier
**Duration of injury**	N/A	0 - 60.8 months, mean = 8.4 months

Supplemental Table 3. Demographics of the RCCL and control groups
